# A new strategy in bioreactor scale-up and process transfer using a dynamic initial vvm according to different aeration pore size

**DOI:** 10.3389/fbioe.2024.1461253

**Published:** 2024-09-10

**Authors:** Huaping Ding, Huanghe Cheng, Jiaxian Wu, Fan Zhang, Can Cao, Siti Aisyah Mualif, Zhenggang Xie

**Affiliations:** ^1^ Department of Biomedical Engineering and Health Sciences, Faculty of Electrical Engineering, Universiti Teknologi Malaysia, Skudai, Johor, Malaysia; ^2^ Dartsbio Pharmaceuticals Ltd., Zhongshan, Guangdong, China; ^3^ T&J Bio-Engineering (Shanghai) Co., Ltd., Shanghai, China; ^4^ Department of Biosciences, Faculty of Sciences, University Technology Malaysia, Johor, Malaysia

**Keywords:** scale-up, cho, aeration pore size, initial ventilation volume, VVM

## Abstract

Monoclonal antibody drugs have grown into a drug category with a market size of over $100 billion since the first product was launched on the market, which naturally creates a large demand for production. At the same time, the $100 billion market is distributed among more than 200 listed drugs, which indicates that the production demand for monoclonal antibody drugs is diverse. To meet this demand, major suppliers offer single-use bioreactors of all sizes. These single-use bioreactors with different specifications, especially the inconsistency of aeration pore sizes, pose great challenges for technology transfer and scale-up production, and the conventional scale-up strategies of constant Power input/volume ratio (P/V) and constant vessel volume per minute (vvm) can no longer meet the needs. This study simplified the selection of technical parameters in bioreactors based on the differences in aeration pore size. Innovatively combined the aeration pore sizes with initial aeration vvm, and comprehensively investigated the relationship between P/V, vvm and aeration pore size by designing experiments (DoE) using the orthogonal test method. The results showed a quantitative relationship between the aeration pore size and the initial aeration vvm in the P/V range of 20 ± 5 W/m^3^. The appropriate initial aeration was between 0.01 and 0.005 m^3^/min for aeration pore size ranging from 1 to 0.3 mm, which was the optimal incubation condition in the bioreactors. The choice of initial ventilation was most related to the final expression. Follow-up studies validated these findings in a 15 L glass bioreactor and a 500 L single-use bioreactor, and the results were consistent with expectations.

## 1 Introduction

Since the first product of monoclonal antibodies was launched in 1986, the global market for monoclonal antibodies has reached $204 billion in 2023, and this market demand is spread across more than two hundred marketed antibodies, as well as thousands of clinically active antibody projects in various stages of clinical research (Crescioli et al., 2024; Zhu et al., 2023). This also signals the need for a wide variety of monoclonal antibody drugs and a huge total production volume. To better suit the needs of such diverse and frequently switching productions of monoclonal antibodies, more and more CDMO companies tend to use single-use bioreactors to increase production capacity (Yoshiura et al., 2024). Currently, single-use bioreactors in the market are mainly controlled by Thermo, Cytia, Sartorius and other major suppliers, and their sizes range from the lowest 50 L to the highest 2,000 L (Jossen et al., 2024) Table 1. The specifications of the single-use bioreactors vary from one supplier to another, especially concerning the aeration aperture size of the drilled-hole sparger (DHS) (Raza et al., 2024). This poses a great challenge for technology transfer between different manufacturers and sizes of single-use bioreactors.

**TABLE 1:** Aeration pore size of single-use bioreactors of different brands and sizes.

Single-use bioreactor (L)	Aeration pore size (mm)
Thermo 50	0.178
Thermo 250	0.233
Thermo 500	0.368
Thermo 2000	0.582
Cytia 50-2000	0.02–1
Sartorius 50-2000	0.5

Nowadays, in the industrial context, most of the technology transfer of monoclonal antibody drugs occurs when there is a need to expand production. For example, when there is a need to use a larger bioreactor that needs to be changed to a new production site. Observing the culture conditions when cell culture is carried out in a bioreactor, except for the two indicators of stirring speed and aeration, the other culture conditions only need to be maintained or scaled up in equal proportions and did not involve the need to recalculate with the expansion of the culture scale (de Mello et al., 2024). At present, P/V and volumetric mass transfer coefficient (*k*_L_a) are mainly used to guide the selection of rotation speed and aeration under the new culture scale. The usual choices would still be based on constant P/V or constant *k*_L_a for both RPM and ventilation conditions at the new culture scale (Xu et al., 2017; Doi et al., 2020). However, observing these two scaling strategies reveals that they are both still flawed. According to the study on the effect of different aeration pore sizes, it can be found that the mass transfer efficiency of oxygen through different aeration pore sizes in the liquid as well as its efficiency in removing carbon dioxide emitted by the cells in the liquid are very different (Berouaken et al., 2023). The differences in oxygen mass transfer efficiency and carbon dioxide removal efficiency cannot be measured using the same scale-up metric. This means that the strategies using constant P/V and constant *k*_L_a are still based on volumetric metrics for calculations on new bioreactors. Although these two strategies take into account most of the hardware differences between the new bioreactor and the original bioreactor, they do not take into account the changes in mass transfer efficiency and the influence of the aeration aperture of the DHS gas distributor, which indicates that there are obvious shortcomings in the current scaling-up strategies (Lim et al., 2023). Meanwhile, it has been reported that the shear force caused by the breaking of bubbles on the culture medium’s surface significantly affects cell growth, which will increase as the bubbles become smaller. Therefore, to ensure cell growth’s safety, the minimum pore size selected in this study was not less than the aeration pore size used in single-use bioreactors.

The effect brought about by the aeration aperture of the DHS should be considered during technology transfer (de Souza Vandenberghe et al., 2022). To examine the impact of aeration aperture size on cell culture, in this study, the selection of culture conditions was simplified, and the culture conditions that needed to be selected were only considered the stirring speed and the initial ventilation volume. The selected rotational speed is sized using P/V and the initial ventilation is sized using vvm. This means only the selection of P/V and initial vvm was considered. A new experimental design was used by combining to simultaneously examine the combined effects of different P/V, vvm, and Pore size conditions on the culture results. It is expected to develop a model that can guide the determination of stirring speed and initial ventilation volume under the condition of using different aeration pore sizes. This model can theoretically be conditioned to provide the appropriate initial ventilation and rotational speed for different aeration pore sizes. Ultimately, it validated the model’s accuracy by using a 0.8 mm aeration pore size in a 15 L bioreactor and a single-use 500 L bioreactor with a 0.368 mm aeration pore size (Thermo Fisher Scientific).

## 2 Materials and methods

### 2.1 Examination of P/V, vvm and aeration pore size effects on the parallel bioreactors

#### 2.1.1 Cell line and culture conditions

In this study, we have used the cell line from the Dartsbio Pharmaceuticals Ltd DS003 project and a stably expressed monoclonal CD-47 antibody cell line was selected. The cell line was monoclonal selected and examined for stability to meet the needs of this experiment. After process optimisation, the culture conditions used for this cell line in 3 L bioreactor (Applikon Bio) culture are shown in the putative table Table 2. In the above experimental conditions, subsequent experiments used the same combinations of basal and recharge media, with the same conditions for recharge volume recharge time and controlled glucose concentration. For this 3 L bioreactor uses a 1 mm aeration pore size, and a vvm of 0.01 m^3^/min (In this study, all vvm conditions were proportional values based on the initial culture volume conversion, to uniformly quantify the relationship between initial ventilation and culture volume. For example, 0.01 times the initial culture volume is the air ventilation volume per min), with a rotation speed corresponding to a P/V of around 15–20 W/m^3^. Subsequent experiments were designed on this basis.

**TABLE 2:** 3 L bioreactor culture conditions for DS003.

Item	Conditions
Basic medium	QuaCell CHO CD04
Feed medium	QuaCell CHO Feed02
Seed density	7 × 10^5^ cells/mL
Volume	Initial volume 1.5 L
T	Initial culture temperature 36.5°C, Day 6 cooled to 35°C
pH	7.0 ± 0.4
DO	50%
Initial air volume	15 mL, 0.01 m^3^/min vvm
Rotation speed	Initial speed 250 rpm, Day 3 set as 300 rpm
Feed amount	Day 3 1%, Day 4–9 2% per day, Day 10–12 2.5% per day, Day 13 2%
Gluc	Control in 4–7 g/L

#### 2.1.2 New bioreactors specification optimization

Parallel bioreactors (T&J Bio-Engineering Shanghai Ltd., CloudReadyTM 500 mL × 24) were used in the experiment to ensure consistency in bioreactor experiments. This parallel reactor uses the same tank with a total volume of 500 mL, a diameter of 70 mm, a height of 130 mm, and a Marine-type paddle with a stirring paddle diameter of 28 mm. A unified software (T&J Bio-Engineering Shanghai Ltd., D2MS) is also used to control these parallel bioreactors. At the same time, the ranges of P/V, vvm and aeration pore size were determined separately. The P/V ranges from 8.8 to 28.8 W/m^3^, and four points, 8.8, 18.8, 23.8 and 28.8 W/m^3^, were selected for examination. Corresponding speeds are 240, 320, 350 and 375 rpm. Aeration pore sizes were examined from 0.3 to 1 mm, and a total of four points, 0.3, 0.5, 0.8, and 1 mm, were selected for examination. This was chosen based on counting common aeration pore sizes in DHS used in various brands of single-use bioreactors. The vvm chose to examine a range from 0.003 to 0.012 m^3^/min, choosing a total of 0.003, 0.0075 and 0.012 m^3^/min points to examine. This was chosen based on the use of 0.01 m^3^/min vvm at 1 mm aeration pore size in a 3 L bioreactor. The working volume of the parallel reactor was 300 mL, and the corresponding air aeration volumes were set to 0.9, 2.25, and 3.6 mL/min. Because of the wide range of conditions, the DoE was used for the experimental design and subsequent analyses. The software used for the experimental design was JMP Pro 17. The experimental protocol is shown in the table below Table 3. A total of 24 experiments in parallel reactors under different conditions need to be completed.

**TABLE 3:** Parallel bioreactor DoE plan.

Id	Aeration pore sizes (*µ*m)	vvm (m^3^/min)	P/V (W/m^3^)
1	500	0.003	18.8
2	500	0.003	28.8
3	800	0.0075	23.8
4	300	0.0075	28.8
5	1,000	0.003	18.8
6	300	0.003	23.8
7	800	0.0075	23.8
8	1,000	0.003	28.8
9	500	0.012	18.8
10	500	0.012	28.8
11	800	0.0075	23.8
12	1,000	0.012	18.8
13	1,000	0.012	28.8
14	300	0.012	23.8
15	800	0.0075	23.8
16	300	0.0075	18.8
17	300	0.003	8.8
18	500	0.012	8.8
19	300	0.012	8.8
20	800	0.0075	8.8
21	500	0.003	8.8
22	1,000	0.003	8.8
23	300	0.0075	8.8
24	1,000	0.012	8.8

#### 2.1.3 Culture process monitoring

Throughout the bioreactor cell culture experiments. The cell growth was monitored using a Cell counter (Thermo Fisher Scientific, countess 3) for cell counting, a Bioprocess analyzer (Roche, Cedex Bio) for metabolic analysis, a Blood-gas analyzer (Radiometer, ABL9) for pCO_2_ and pO_2_ monitoring. All of the above assays are designed to monitor cell growth status, determine cell growth status and control glucose concentration. Finally to uniformly collect samples at the end of the culture on day 14 using Protein A-HPLC method to analyse the final titer.

#### 2.1.4 Data analysis

After all the data have been collected, mainly the final titer results, a 3D scatter analysis has been done using the JMP software to speculate on the P/V and initial vvm combinations best suited to achieve the highest titer at different aeration pore sizes. The only basis for judging the goodness of the results obtained using different conditions is the final titer.

### 2.2 Validation experiment on 15 L bioreactor

#### 2.2.1 Cell line and culture conditions

In conjunction with the results of the DoE test, the cell line by the Dartsbio Pharmaceuticals Ltd. DS006 project was selected for validation experiments on a 15 L bioreactor (Thermo Fisher Scientific). The basal and feed media used for this cell line were the same as DS003, QuaCell CHO CD04, and QuaCell CHO Feed02. The total feed amount was 27% of the initial culture volume, with 3%, 4.5%, 5%, 5%, 5%, and 119 4.5% feed on days 3, 5, 7, 9, 11, and 13. The seeding density of 7.5 × 10^5^ cells/mL. The culture temperature was initially 36.5°C and was cooled down to 33°C on the sixth day. Test the glucose content after the feed is completed, calculate the glucose supplementation according to the test results, and feed the glucose concentration to 6–8 g/L.

Three rounds of comparative experiments under different conditions were conducted to verify the optimal ventilation conditions when using a 0.8 mm aeration pore size. The detailed experimental conditions are shown in the table below Table 4. In the three comparison experiments, the first and second experiments used ventilation of 0.01 m^3^/min vvm, the second experiment initial P/V was reduced from 20 to 18 W/m^3^ for the first experiment, and the third experiment used an initial P/V of 18 W/m^3^ and a vvm of 0.0089 m^3^/min. Subsequent oxygen delivery is controlled by setting DO to 40%. Test the glucose content after the replenishment is completed, calculate the glucose supplementation according to the test results, and replenish the glucose concentration to.

**TABLE 4:** Comparison of 15 L bioreactor culture conditions.

Bioreactor	15 L-B1	15 L-B2	15 L-B3
Initial culture volume	9 L	9 L	9 L
Seeding density	7.5 × 10^5^ cells/mL	7.5 × 10^5^ cells/mL	7.5 × 10^5^ cells/mL
Feeding strategy	Day 3, 5, 7, 9, 11, 13	Day 3, 5, 7, 9, 11, 13	Day 3, 5, 7, 9, 11, 13
Culture temperature	D0: 36.5°C, D6: 33°C	D0: 36.5°C, D6: 33°C	D0: 36.5°C, D6: 33°C
pH	D0–D14: 7.0 ± 0.3	D0–D14: 7.0 ± 0.3	D0–D14: 7.0 ± 0.3
DO control	40%	40%	40%
Rotate speed	130 rpm	D0: 125 rpm, D3: 130 rpm	D0: 125 rpm, D3: 130 rpm
Aeration strategy	Air 90 mL/min (0.8 mm)	Air 90 mL/min (0.8 mm)	Air 80 mL/min (0.8 mm)
Glucose	6–8 g/L	6–8 g/L	6–8 g/L

#### 2.2.2 Culture process monitoring

In these bioreactor cell culture experiments, cell counting was performed using a Cell counter (Countstar, IC1000) for cell counting, a Bioprocess analyzer (Sieman, M-900) for metabolic analysis, a Blood-gas analyzer (Sieman, G-100) for pCO_2_ and pO_2_ monitoring, and finally to collect samples at the end of the culture on day 14 using Protein A - HPLC method to analyse the final titer. A final observation was made to see which of the conditioned cultures used in the bioreactors gave final titer results consistent with those in the shake flasks. The suitability of the conditions chosen in the bioreactor for cell growth was judged by comparing the results of the bioreactor with those of the shake flask culture.

#### 2.2.3 15 L bioreactor culture solution quality analysis

To better confirm the differences brought about by the use of different P/V and vvm conditions in the 15 L bioreactor, the supernatant obtained from the culture was purified using affinity chromatography to obtain the target antibody, which was analysed for size-exclusion chromatography (SEC) and capillary electrophoresis (CE) purity.

The equipment used to purify the culture solution was an AKTA Avant25 and the affinity chromatography used was Pro chievA (Avantor), after the system was prepared the column was equilibrated with PBS for 5 CV, followed by a sampling of 115 mL, followed by 4 CV rinse with PBS + 0.1% TritonX-114, followed by 4 CV rinse with PBS + 0.5 M NaCl, then equilibrated with PBS by 4 CV, and finally eluted with 0.1 M HAc pH 4.1, collecting a UV count of 200 mAu-Peak-200 mAu.

SEC analyses were performed using H-Class plus Bio (Waters) and BioCore SEC - 300 (NanoChrom) with a sample volume of 10 µL and an equilibrium solution of 50 mM PB + 300 mM NaCl pH 6.8 at an equilibrium flow rate of 0.25 mL/min.

CE purity was measured using Mautice (Protein Simple) and the CE-SDS PLUS Size Application Kit reagent. The sample concentration was adjusted to 0.2–1 mg/mL using 1 × PLUS Sample Buffer, and the salt concentration was less than 50 mM. 50 *µ*L of the sample was taken into a 1.5 mL EP tube, and 1 *µ*L of 25 × Internal Standard were added and vortexed to mix, and finally, 2.5 µL of 250 mM iodoacetamide was added and vortexed to mix. Remove 50 µL of processed sample in a 96-well plate and assay using the standard kit method.

### 2.3 Validation experiment on 500 L single-use bioreactor

#### 2.3.1 Cell line and culture conditions

In conjunction with the results of the DoE test, the cell line by the Dartsbio Pharmaceuticals Ltd. DS002 project was selected for validation experiments on a 500 L single-use bioreactor (Thermo Fisher Scientific). The basal and feed media used for this cell line were not the same as DS003, they are DS002 project customised base medium and feed medium. The composition of those two mediums differs from that of the medium used before in only two components. The total feed amount (Based on initial working volume) was 22%, with 2%, 4%, 4%, 4%, 4%, 4%, 4%, feed on days 3, 4, 6, 8, 10, and 12. The culture temperature was initially 36.5°C and was cooled down to 34°C on the fourth day. Supplement the glucose level to 4–7 g/L after the feed.

Based on the same culture conditions as shake flasks above, in this 500 L bioreactor with a DHS pore size of 0.368 mm, an initial culture volume of 350 L and a seeding density of 7.5 × 105 cells/mL. The initial rotation speed was set at 85 rpm and was adjusted to 95 rpm after refilling on the third day. Based on the volumetric accounting the P/V was controlled at 16–20 W/m^3^ (P/V value because of the feed, the working volume increases, in the case of the rotational speed is constant will gradually decline) throughout the whole culture time. The initial ventilation was set at 2 L/min, which was converted to vvm of 0.0057 m^3^/min. Subsequent oxygen delivery is controlled by setting DO to 40%.

#### 2.3.2 Culture process monitoring

In these bioreactor cell culture experiments, it was planned to use a Cell counter (Beckman, Vi-Cell) for cell counting, a Bioprocess analyzer (Roche, Cedex Bio) for metabolic analysis, a Blood-gas analyzer (Siemens, Diagnostics 348) for pCO2 and pO2 monitoring, and finally to collect samples at the end of the culture on day 14 using Protein A-HPLC method to analyse the final titer. A final observation was made to see which of the conditioned cultures used in the bioreactors gave final titer results consistent with those in the shake flasks.

#### 2.3.3 Data analysis

This batch of cell culture has a huge sample volume. It was subsequently undergo a three-step chromatographic purification process on an industrial scale. These samples will be used in clinical trials. The final sample purity data obtained was very high, making it unsuitable for quality data comparisons. Because the purity here is obtained through fine purification. For this reason, production on this 500 L scale single-use bioreactor was compared to shake flask culture for cell density, cell viability, and titer. Observe whether the cell status throughout the culture process is consistent with the data on shake flask culture.

## 3 Results

### 3.1 DoE experiments for use orthogonal test method examines the effect between P/V, vvm and aeration pore size

**FIGURE 1:**
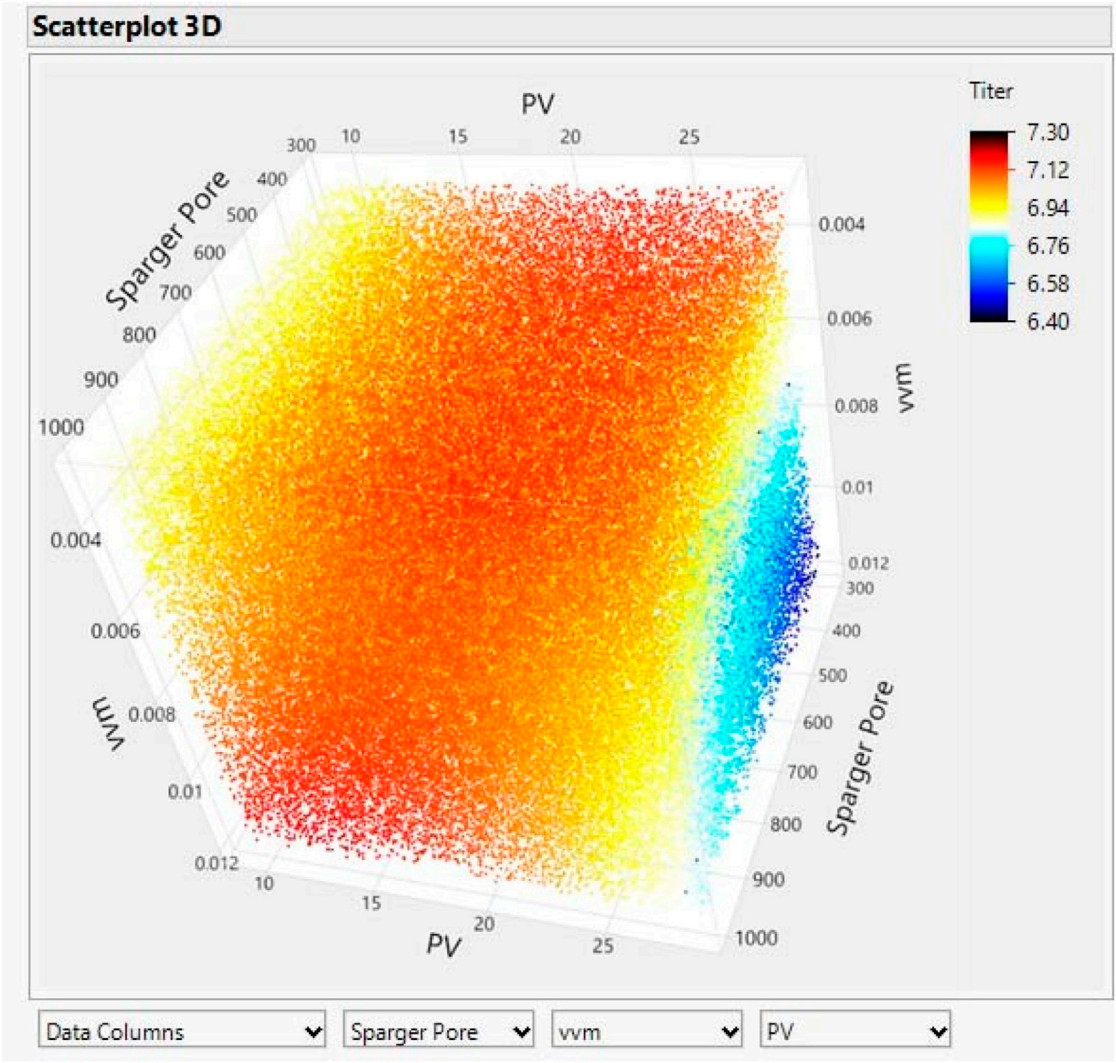
Results of 3D scatter analysis of DoE experimental results.

The final titer results for the 24 parallel bioreactors are shown below Table 5. Based on the above results 3D scatter analysis using JMP software, a range of P/V and initial vvm appropriate for a particular aeration pore size can be observed based on the colour distribution. This graph was created by comprehensive evaluation and prediction of the experimental results using JMP software Figure 1. It is a predictive graph that incorporates real data. The darker red colour in the graph represents a range of conditions with higher yields. Based on the colour distribution, it can be seen that the P/V conditions for higher yields are between 15 and 25 W/m^3^ for different aeration apertures, with higher P/V being detrimental to expression, especially when initial vvm is also selected to be higher at the same time. As the aeration aperture size goes from 1 to 0.3 mm, the range of suitable initial vvm decreases gradually from 0.01 to 0.005 m^3^/min, showing an equivalently smaller relationship. Along with reducing the aeration aperture size, the appropriate initial vvm conditions show a curved decreasing style, with an arc visible along the way. Meanwhile, statistical analysis of the experimental results using JMP software reveals that the *P*-value results show that the most relevant parameter to titer is vvm, followed by aeration aperture size, and P/V is at the end.

**TABLE 5:** Parallel bioreactor DoE results.

Id	Aeration pore sizes (*µ*m)	vvm (m^3^/min)	P/V (W/m^3^)	Titer-HPLC (g/L)
1	500	0.003	18.8	7.07
2	500	0.003	28.8	7.08
3	800	0.0075	23.8	7.06
4	300	0.0075	28.8	6.79
5	1,000	0.003	18.8	7.05
6	300	0.003	23.8	6.84
7	800	0.0075	23.8	7.06
8	1,000	0.003	28.8	6.88
9	500	0.012	18.8	6.68
10	500	0.012	28.8	6.52
11	800	0.0075	23.8	6.75
12	1,000	0.012	18.8	6.94
13	1,000	0.012	28.8	6.66
14	300	0.012	23.8	6.57
15	800	0.0075	23.8	6.93
16	300	0.0075	18.8	6.66
17	300	0.003	8.8	6.67
18	500	0.012	8.8	6.71
19	300	0.012	8.8	6.80
20	800	0.0075	8.8	6.81
21	500	0.003	8.8	6.66
22	1,000	0.003	8.8	6.57
23	300	0.0075	8.8	6.81
24	1,000	0.012	8.8	6.97

### 3.2 Validation experiment on 15 L bioreactor

**FIGURE 2:**
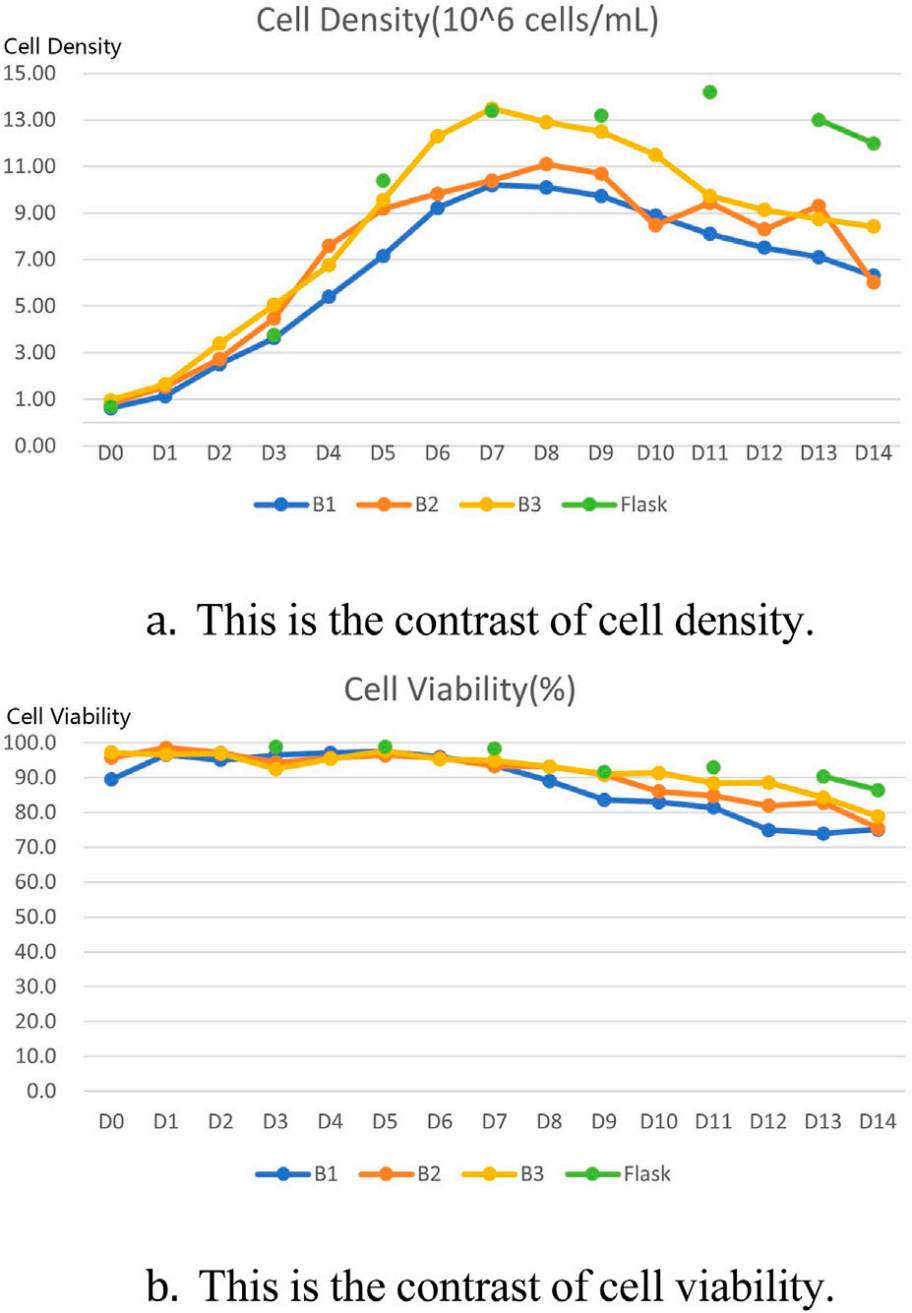
Comparison of 15 L bioreactor culture results with shake flask culture results. **(A)** Comparison of cell density between three rounds of 15 L bioreactor culture and shake flask culture. **(B)** Comparison of cell viability between three rounds of 15 L bioreactor culture and shake flask culture.

The titer results of comparative tests using different conditions in a 15 L bioreactor are shown in the table below Table 6. Data on cell density, cell viability, sugar and lactate metabolism during culture are shown below Figure 2. Comparing the data, it can be seen that optimising only the P/V conditions does not work well to obtain the desired results, whereas optimising the parameters of vvm achieves results that are consistent with the performance of shake flasks in a 15 L bioreactor. The results of the quality analyses showed that the adjustment of P/V and vvm conditions had little effect on the purity results Table 7.

**TABLE 6:** 15 L bioreactor test titer results.

Item	B1	B2	B3	Flask
Initial volume	9 L	9 L	9 L	
Rotate speed	130 rpm	125 rpm	125 rpm	
Initial P/V	20.86 W/m^3^	18.54 W/m^3^	18.54 W/m^3^	
Initial air	90 mL/min	90 mL/min	80 mL/min	
Vvm	0.01 m^3^/min	0.01 m^3^/min	0.0089 m^3^/min	
Pore size	0.8 mm	0.8 mm	0.8 mm	
Titer	2.30 g/L	2.43 g/L	3.84 g/L	3.7 g/L

**TABLE 7:** 15 L bioreactor quality analysis results.

Item	SEC (%)	CE (%)
B1	93.90	92.6
B2	91.95	93.4
B3	94.63	

### 3.3 Validation experiment on 500 L single-use bioreactor

**FIGURE 3:**
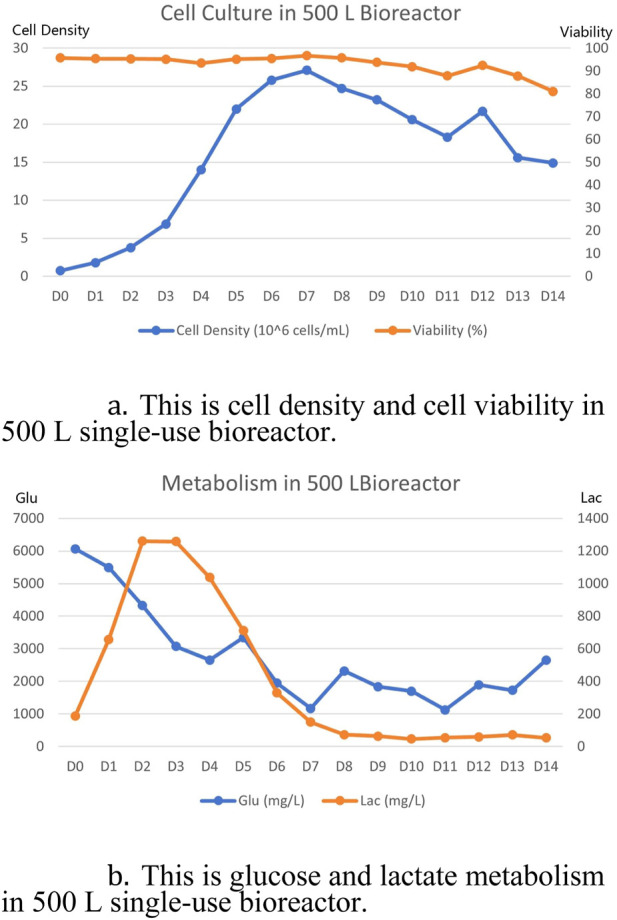
500 L single-use bioreactor culture results. **(A)** Cell density and cell viability results of 500 L single-use bioreactor culture. **(B)** Glucose and lactate metabolism results of 500 L single-use bioreactor culture.

Detailed culture data is shown in the chart below Figure 3. The final titer at harvest on day 14 was 5.7 g/L. The cell viability at the last day of harvest was still maintained at about 80% and the cell density was maintained at 1.5 × 10^7^ cells/mL. Lactate metabolism was well depleted in subsequent cultures after the logarithmic growth period had accumulated, consistent with the conventional culture cycle. Overall, the entire culture cycle was as expected, as was the final expression.

## 4 Discussion

From the birth of monoclonal antibody drugs to the present day, more and more studies have been conducted for the scale-up culture of bioreactors, and two important guiding principles, constant P/V and constant *k*_L_a scale-up, have been developed. However, as mentioned earlier, these two scale-up strategies still have obvious shortcomings (Jaibiba et al., 2020). Recently, studies have also been conducted to investigate scale-up under the conditions of both constant P/V and vvm (Kreitmayer et al., 2023). However, using the same vvm at different DHS pore sizes is still a flawed scale-up strategy according to the gas mass transfer characteristics, and the effect of DHS pore size must be taken into account during technology transfer or scale-up cell culture (Zhong et al., 2022; Bernemann et al., 2024).

The impact of DHS pore size is mainly seen in the effect on the efficiency of oxygen mass transfer, a culture nutrient, and the removal of carbon dioxide, a culture metabolite (Tran et al., 2023). It has been shown that when using smaller DHS pore sizes, the increase in specific surface area due to smaller bubbles as well as changes in gas flow rates enhances the efficiency of oxygen mass transfer (McAndrew and Kauffman, 2021). Oxygen demand should be consistent during cell growth, which inevitably leads to a reduction in oxygen aeration during subsequent cell culture when smaller DHS pore sizes are used. At the same time, the dissolution rate of carbon dioxide in water is much higher than that of oxygen, and the reduction of aeration volume inevitably reduces the efficiency of carbon dioxide removal, inducing an increase in the partial pressure of carbon dioxide, which is unfavourable to the results of the culture (Ohde et al., 2021). A study using a fixed *k*_L_a (CO_2_)/*k*_L_a (O_2_) ratio in 200 and 2,000 L bioreactors to guide the scale-up showed consistent results (Carpio, 2020). This also shows that the effect of the DHS pore size on mass transfer is necessary to be considered, as it has different effects on the *k*_L_a of oxygen and carbon dioxide, which must be balanced to ensure successful technology transfer or scale-up of the culture.

In this study, the selection of culture conditions on the bioreactor was simplified by determining only the rotational speed and initial aeration based on two indicators, P/V and initial vvm, respectively, under a defined aeration aperture size, and examining the relationship between the three variables P/V, initial vvm, and pore size. In this way, during the whole cell culture process, under a fixed pore size, the device autonomously decides the oxygen ventilation according to the oxygen consumption (DO), and the change of oxygen mass transfer brought by the DHs pore size was taken into account. At the same time, different aeration pore sizes have different initial aeration volumes to balance the efficiency of carbon dioxide removal, which was a good way to find the right set of conditions in the bioreactor to build a suitable environment for the growth of CHO cells and to achieve consistent results from shake flasks to larger scale bioreactors. A recommended range of P/V and initial aeration for a given aeration aperture size can be obtained. This recommendation can be validated in larger-scale bioreactors. Through the above experimental design, firstly, it was expected to determine how much different aeration pore sizes affect the cell culture results when using single-use bioreactors with different hardware specifications. Then secondly, It was hoped to establish a new scale-up model that can be used to achieve stable scale-up by using different initial vvm determined by different aeration pore sizes while using a constant P/V strategy when transferring or scaling up processes in different bioreactors.

Based on the experimental results, it was found that the selection of the initial vvm was important for the experimental results, and the selection of the initial vvm was correlated with the aeration pore sizes, which confirms that the effect of the aeration pore sizes on the experimental results was present and strong. Also based on the experimental results the proposed scale-up model is that the constant P/V range of 15–25 W/m^3^ is recommended to be used and the initial vvm is selected according to the size of the aeration pore sizes. This result was also validated on a 15 L glass bioreactor and a 500 L single-use bioreactor, the desired results were obtained.

## 5 Conclusion

Based on the above experimental data, it can be observed that the optimal range of values for the selection of P/V shown in the DoE experiments is in the upper and lower 20 W/m^3^ when cell culture is performed in the bioreactor. Under this range of P/V, it can also be observed that along with the change in aeration pore size, when the aeration pore size is smaller, more consistent culture results can be obtained by selecting a lower vvm value than using a larger aeration pore size for the cell culture, with a recommended range of vvm 0.01 to 0.005 m^3^/min. Finally, validation experiments in a 15 L bioreactor also demonstrated that using a P/V of 18 W/m^3^ and a vvm of 0.0089 m^3^/min gave consistent results with cell culture in shake flasks. It was also found that when using a 0.8 mm aeration pore in the 15 L bioreactor, comparing the results using a vvm of 0.01 and 0.0089 m^3^/min, it was found that the results using 0.0089 m^3^/min were consistent with culturing in a shake flask. In a 500 L single-use bioreactor, an initial vvm of 0.0057 m^3^/min was chosen when the aeration pore size was 0.368 mm, resulting in titer consistent with shake flask. All of this is also consistent with the speculation of the DoE experiment. These two validation experiments were conducted only once to verify the reliability of the model, for the reason that the cost in those experiments was very higher. The model needs further research and validation. In conclusion, it is clear from the above experiments that the change in aeration pore size should be considered as an important factor when transferring or scaling up processes in different bioreactors, and it is recommended that different initial vvm be used to match the change in aeration pore size to obtain stable cell culture results.

## Data Availability

The original contributions presented in the study are included in the article/Supplementary Material, further inquiries can be directed to the corresponding author.
