# Supplementary material for: A new strategy in bioreactor scale-up and process transfer using a dynamic initial vvm according to different aeration pore size
Source: Front Bioeng Biotechnol. 2024 Sep 10;12:1461253. doi: 10.3389/fbioe.2024.1461253 (PMC11420042; doi:10.3389/fbioe.2024.1461253)

Fit Group

Response Titer试剂盒

Actual by Predicted Plot

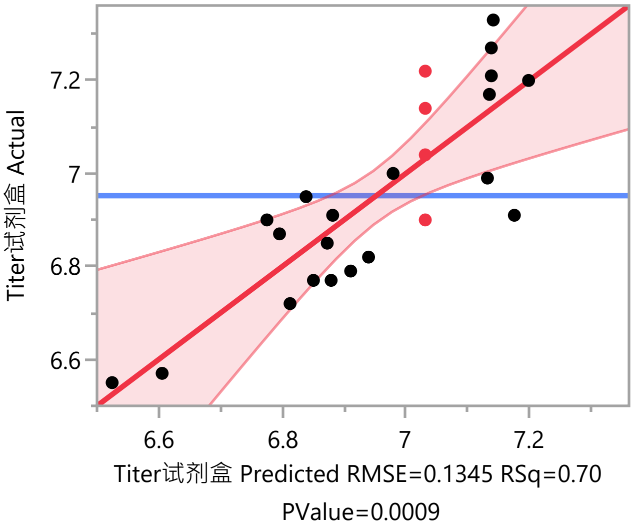

Effect Summary

Source	Logworth	PValue	
vvm(0.003,0.012)	3.447	0.00036	
vvm*PV	2.901	0.00126	
Sparger Pore(300,1000)	1.926	0.01186	
Sparger Pore*vvm	1.673	0.02122	
PV*PV	1.241	0.05747	
PV(18.8,28.8)	1.192	0.06427	^

Lack Of Fit

Source	DF	Sum of Squares	Mean Square	F Ratio
Lack Of Fit	14	0.25057699	0.017898	0.9404
Pure Error	3	0.05710000	0.019033	**Prob > F**
Total Error	17	0.30767699		0.6040
				**Max RSq**
				0.9449

Residual by Predicted Plot

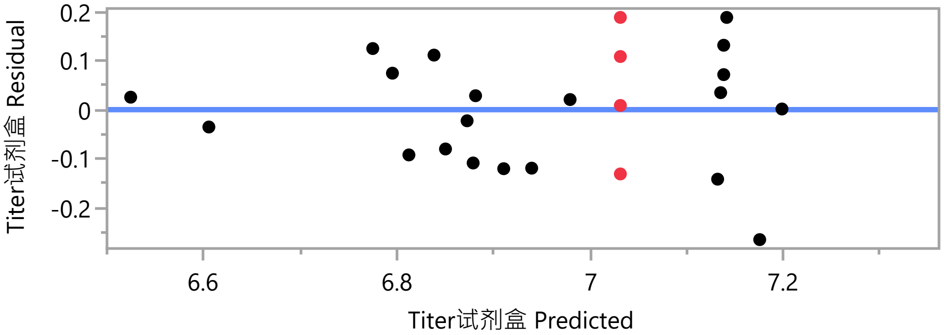

Fit Group

Response Titer试剂盒

Studentized Residuals

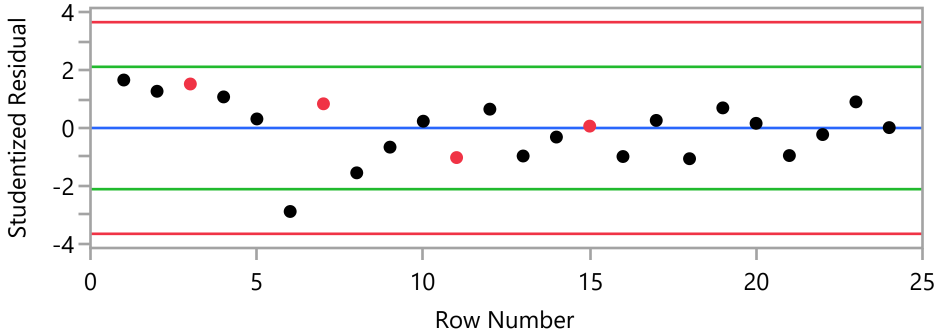

Externally studentized residuals with 95% simultaneous limits (Bonferroni) in red, individual limits in green.

Prediction Profiler

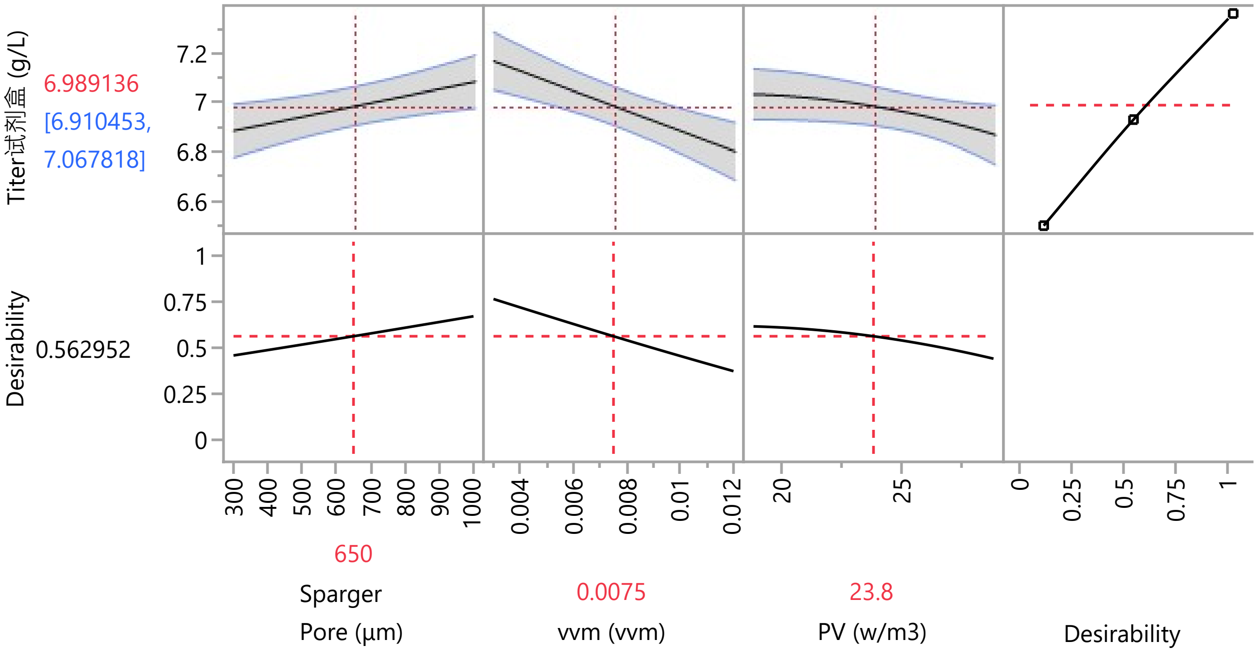

Response PVCD

Actual by Predicted Plot

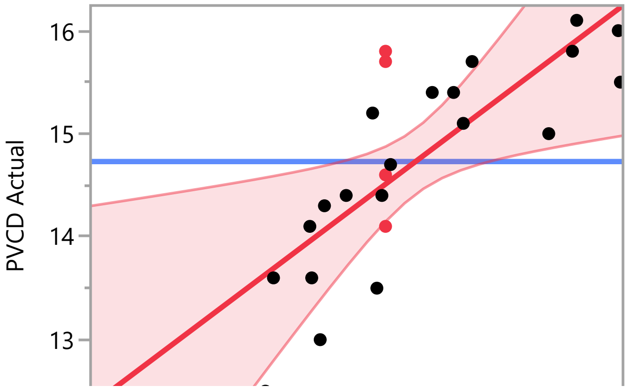

Fit Group

Response PVCD

Actual by Predicted Plot

PVCD Predicted RMSE=0.7386 RSq=0.59

PValue=0.0096

Effect Summary

Source	Logworth	PValue	
PV(18.8,28.8)	2.452	0.00353	
PV*PV	2.187	0.00650	
Sparger Pore*PV	1.972	0.01067	
Sparger Pore*vvm	1.186	0.06523	
vvm(0.003,0.012)	0.437	0.36545	^
Sparger Pore(300,1000)	0.426	0.37504	^

Lack Of Fit

Source	DF	Sum of Squares	Mean Square	F Ratio
Lack Of Fit	14	7.1849944	0.513214	0.7367
Pure Error	3	2.0900000	0.696667	**Prob > F**
Total Error	17	9.2749944		0.7038
				**Max RSq**
				0.9085

Residual by Predicted Plot

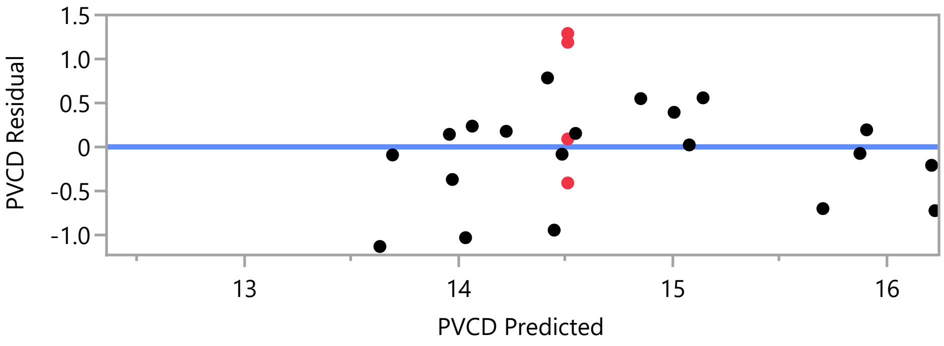

Studentized Residuals

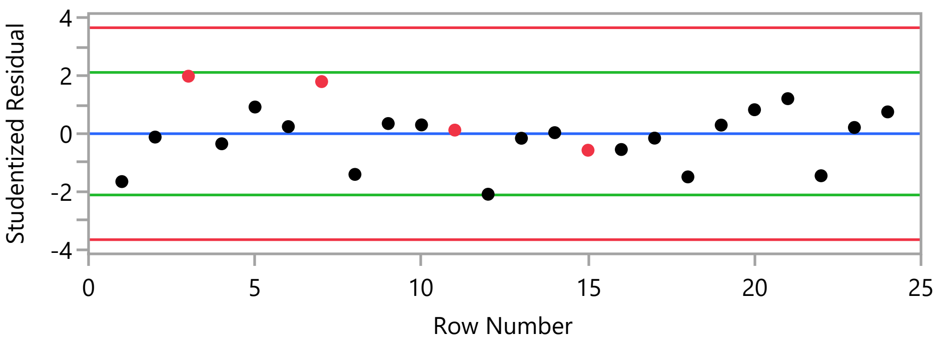

Fit Group

Response PVCD

Studentized Residuals

Externally studentized residuals with 95% simultaneous limits (Bonferroni) in red, individual limits in green.

Prediction Profiler

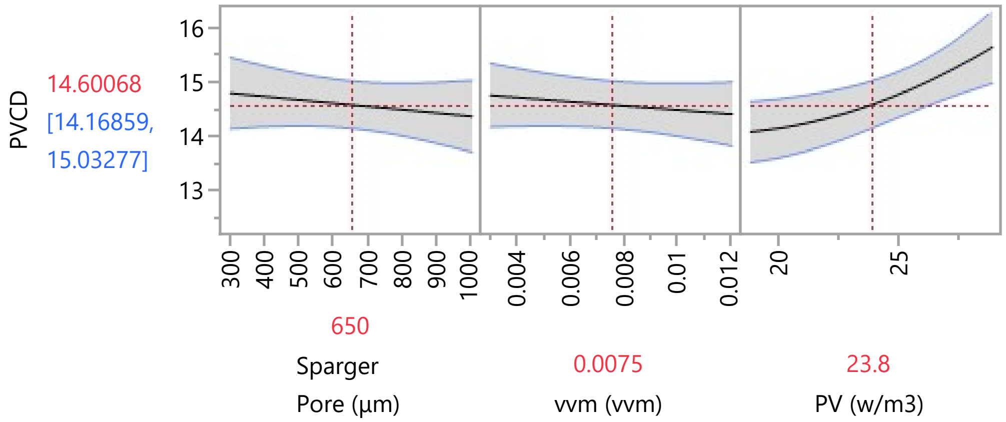

Response Viability

Actual by Predicted Plot

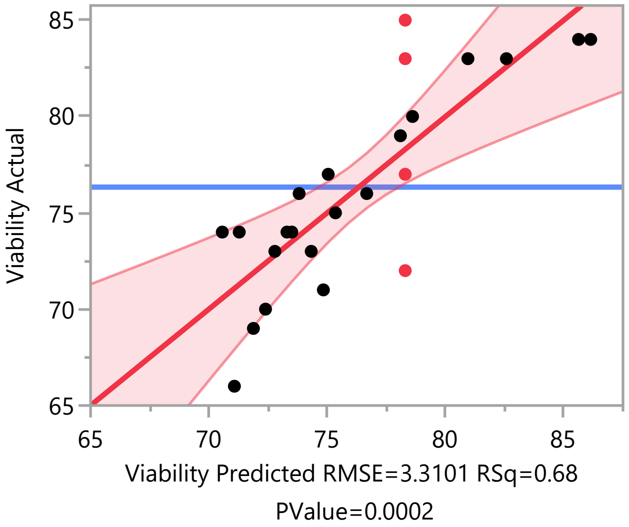

Fit Group

Response Viability

Effect Summary

Source	Logworth		PValue
PV(18.8,28.8)	3.999		0.00010
vvm(0.003,0.012)	2.853		0.00140
vvm*PV	2.301		0.00500
Sparger Pore(300,1000)	0.166		0.68203

Lack Of Fit

Source	DF	Sum of Squares	Mean Square	F Ratio
Lack Of Fit	16	103.43166	6.4645	0.1851
Pure Error	3	104.75000	34.9167	**Prob > F**
Total Error	19	208.18166		0.9907
				**Max RSq**
				0.8367

Residual by Predicted Plot

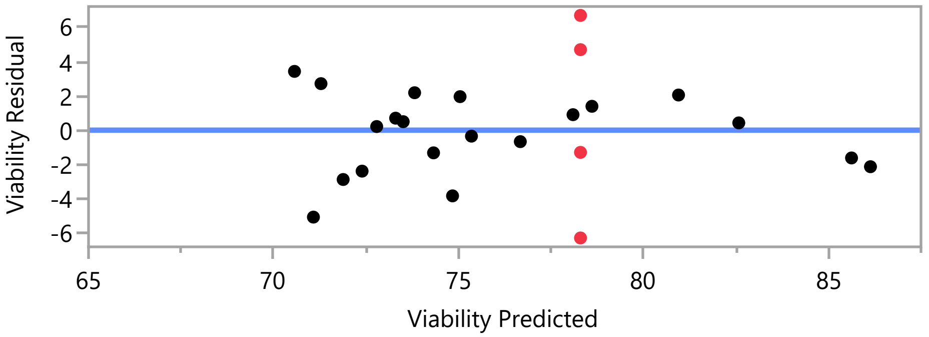

Studentized Residuals

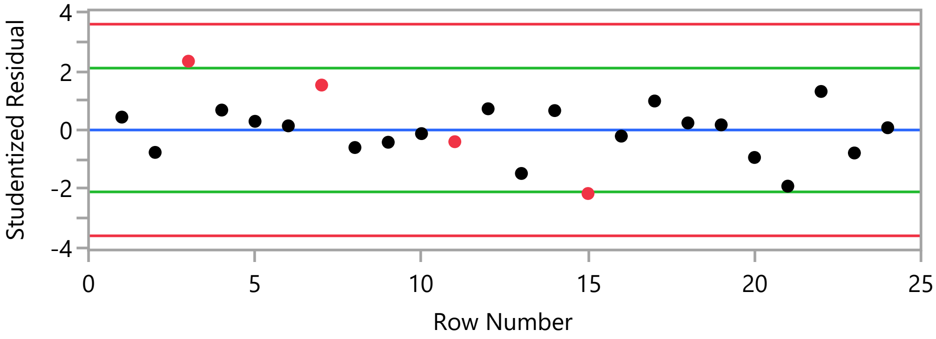

Externally studentized residuals with 95% simultaneous limits (Bonferroni) in red, individual limits in green.

Fit Group

Response Viability

Prediction Profiler

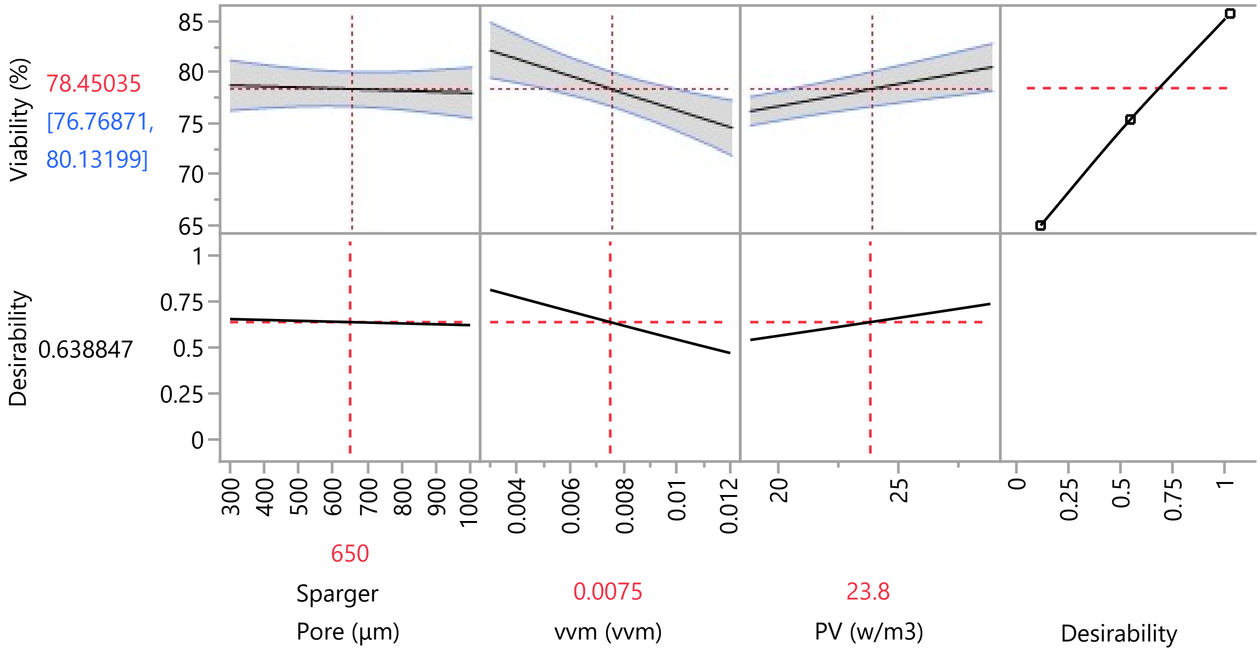

Prediction Profiler

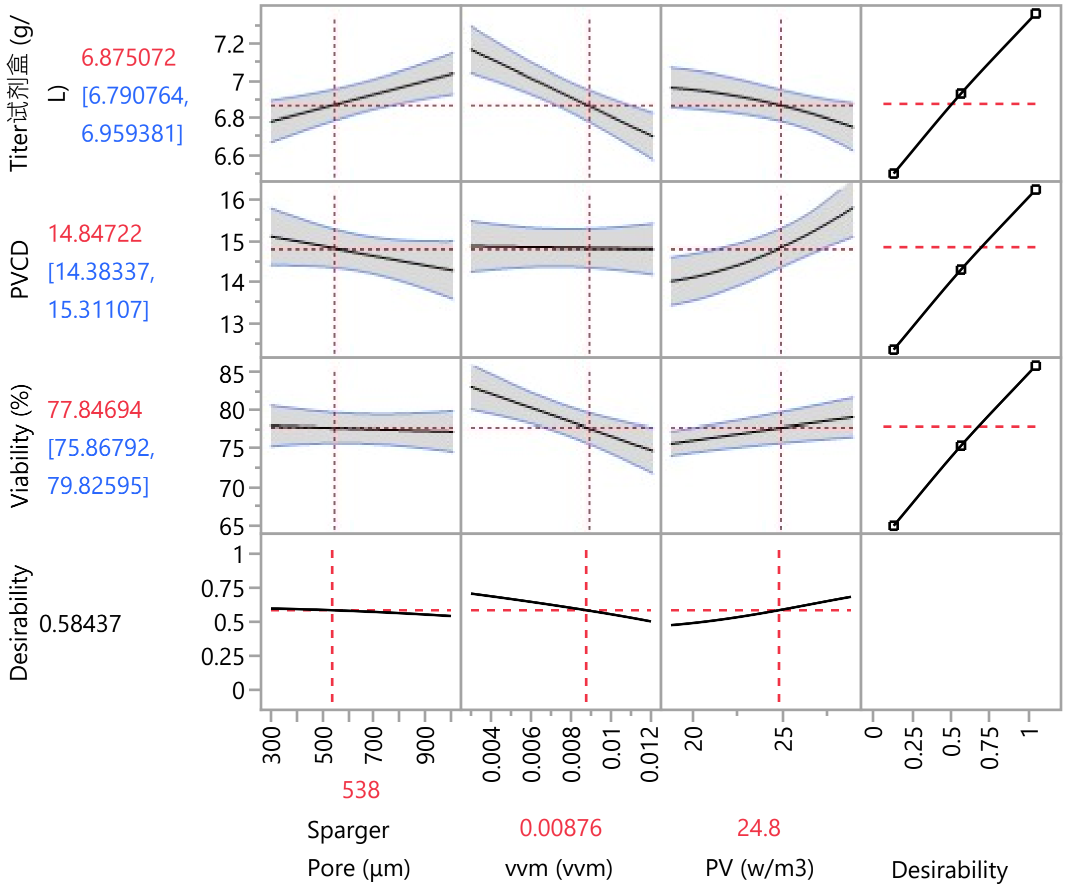

Supplement: Supplementary file 1 [file DataSheet1.PDF]
